# Disability Weights for Global Burden Estimation of Orofacial Pain

**DOI:** 10.1177/00220345251363852

**Published:** 2025-09-15

**Authors:** A. Lövgren, P. Liv, J. Durham, D.A.G. Goncalves, F.P. Kapos, S.F. Kothari, M. Drangsholt, C.C. Peck, C.M. Visscher, L. Ong, P. Svensson

**Affiliations:** 1 Department of Odontology, Orofacial Pain and Jaw Function, Faculty of Medicine, Umeå University, Umeå, Sweden; 2 Department of Public Health and Clinical Medicine, Faculty of Medicine, Umeå University, Umeå, Sweden; 3 School of Dental Sciences, Newcastle University, Newcastle upon Tyne, United Kingdom; 4 Newcastle Hospitals NHS Foundation Trust, Newcastle upon Tyne, United Kingdom; 5 School of Dentistry, São Paulo State University, Araraquara, Brazil; 6 Department of Orthopaedic Surgery and Duke Clinical Research Institute, School of Medicine, Duke University, Durham, NC, United States; 7 Department of Psychology and Behavioural Sciences, School of Business and Social Sciences, Aarhus University, Aarhus, Denmark; 8 Hammel Neurorehabilitation Centre and University Research Clinic, Department of Clinical Medicine, Aarhus University, Hammel, Denmark; 9 Department of Oral Medicine, University of Washington, Seattle, WA, USA; 10 Faculty of Dentistry, National University of Singapore, Singapore; 11 Department of Orofacial Pain and Dysfunction, Academic Centre for Dentistry Amsterdam, University of Amsterdam and Vrije Universiteit Amsterdam, Amsterdam, the Netherlands; 12 Institute for Health Metrics and Evaluation, University of Washington, Seattle, WA, USA

**Keywords:** epidemiology, facial pain, modeling, public health, temporomandibular disorders, quality of life

## Abstract

The global burden of orofacial pain (OFP), including temporomandibular disorders (TMDs), has never been estimated due to lacking disability weights (DWs). This is a significant limitation in the World Health Organization’s goals for oral health. The present study presents DWs with directions for the development of corresponding health state descriptions. We used general population data on OFP and TMD (*n* > 180,000) with linked data on health-related quality of life from the Short Form Health Survey (SF-36; *n* > 110,000). From the SF-36 sum scores, a cumulative DW was mapped, and condition-specific DWs adjusted for common risk factors were calculated. Pain disability and related self-reported clinical characteristics were presented descriptively in a subsample of 300 individuals. In the study sample (*n* = 26,253, 49.9% women), participants with OFP reported significantly lower scores on the mental and physical components of the SF-36 when compared with pain-free individuals (*P* < 0.001), as did individuals with TMD as compared with those without TMD (*P* < 0.001). After adjusting for the presence of common health states, the mean DW was 0.024 (95% CI, 0.011 to 0.038) for OFP and 0.026 (95% CI, 0.016 to 0.038) for TMD. Individuals with higher pain disability reported higher pain intensity as well as increased pain catastrophizing and functional jaw limitations. Our findings demonstrate a feasible solution for estimating DW for OFP and TMD as an important step toward incorporation of these conditions into the Global Burden of Disease study and suggest a greater impact of pain than other common oral diseases. Further efforts are needed to develop lay descriptions and validate DW findings in other populations.

## Introduction

Orofacial pain (OFP) is one of the most common reasons for people to seek dental care (John et al. 2020). On a societal level, chronic OFP incurs substantial costs, estimated, for example, at £701 billion to £1 trillion in annual health care costs worldwide for women aged 20 to 39 y alone (Durham 2023). Indirect costs for interference with daily activities, including absence from or presenteeism at work, are also substantial, estimated at £2,421 to £3,510 per individual per annum (Breckons et al. 2018; Salinas Fredricson et al. 2023; Vallin et al. 2024). Despite this, patients with OFP often go underdetected and undertreated in health care, which compounds the inequities for this already vulnerable group (Lovgren et al. 2018). OFP is often excluded from general medical systems and policies (Slade and Durham 2020). For example, despite the inclusion of less prevalent pain conditions and other oral diseases (e.g., caries, periodontal disease, and edentulism), OFP is not considered in the Global Burden of Disease assessment (GBD 2019 Diseases and Injuries Collaborators 2020; GBD 2021 Low Back Pain Collaborators 2023). This grave omission hinders discussions with policy makers regarding reorganizing OFP management at community and individual levels. Therefore, incorporating global measures of OFP is a much-needed and necessary next step to improve all aspects of oral health.

The Global Burden of Disease initiative began in 1990 to quantify the loss of a period of healthy life across different regions and periods. The global burden of a nonmortal condition is estimated by a combination of the condition’s prevalence and its disability weight (DW). For various mutually exclusive states of health, a DW is assigned in a range from 0 (full health) to 1 (death; Charalampous et al. 2022). At present, DWs are available for >2,100 conditions (Global Burden of Disease Collaborative Network 2020). In addition, each health state and its associated symptoms should be presented with lay descriptions to ensure accessibility and understanding for diverse audiences. Therefore, estimating the global burden of OFP requires valid DWs with accepted health state descriptions. Our first aim was to propose DWs for OFP, including temporomandibular disorders (TMDs). Second, we aimed to provide directions for the development of corresponding lay descriptions for OFP/TMD states.

## Methods

### Study Design and Study Population

This cross-sectional study was based on 3Q/TMD data from the general population of Västerbotten, Northern Sweden. The data were obtained from routine visits to the Public Dental Health Services from May 2010 to December 2017 (*n* = 525,707 visits, *n* = 180,308 unique individuals; equal gender distribution). This database was linked to registry data from the Västerbotten Intervention Programme (VIP; registry data, *n* > 100,000; data collection ongoing), including information on medical status, lifestyle factors, and health-related quality of life (Norberg et al. 2010).

Clinical examination data were collected from a subsample from a Public Dental Health Services cohort to gain descriptive information about its states of health. This cohort consisted of 300 participants (202 women) aged 20 to 69 y (mean age: 3Q positives, 38.3 y [SD, 13.9]; 3Q negatives, 39.1 y [SD, 13.6]). Details on the recruitment process, study design, and sample characteristics have been published (Lövgren et al. 2016). These participants underwent a standardized clinical examination following the Diagnostic Criteria for Temporomandibular Disorders (DC/TMD) protocol (Schiffman et al. 2014) and completed a set of questionnaires for self-reported clinical status from the DC/TMD axis II, including the Graded Chronic Pain Scale (GCPS) 1-mo version (Von Korff et al. 1992; Sharma et al. 2021) and the Jaw Functional Limitation Scale–20 (JFLS-20; Ohrbach et al. 2008). In addition, respondents answered the Pain Catastrophizing Scale (Pedler 2010), the Oral Behavior Checklist (Kaplan and Ohrbach 2016), and a single question on sleep quality.

### Assessment of Health-Related Disability

There are at least 5 data-driven methods for calculating DW (Charalampous et al. 2022). One of the accepted methods for calculating DW is by estimation from the Short Form Health Survey (SF-36), a generic questionnaire on health-related quality of life used to compare disease burdens (Ware and Sherbourne 1992). The SF-36 evaluates 2 main components—the Physical Component Summary (PCS) and the Mental Component Summary (MCS)—across 8 dimensions derived from 36 items. Each dimension and both constructs are scored from 0 to 100, with higher scores indicating better health-related quality of life. The SF-36 scores can be replicated in SF-12 scores (Jenkinson et al. 1997). PCS and MCS from the SF-36 were obtained from the linked VIP registry data.

### Explanatory Variables for DWs

For each recorded visit at the Public Dental Health Services, the status for each of the 3 states—OFP, jaw catching/locking, and TMD—was based on answers to 3 validated screening questions (3Q/TMD) with a dichotomous yes/no response (Lövgren et al. 2016):

Q1: Do you have pain in your temple, face, jaw, or jaw joint, once a week or more?
Q2: Do you have pain when you open your mouth or chew, once a week or more?
Q3: Does your jaw lock or become stuck, once a week or more?

OFP was defined as an affirmative answer to Q1 or Q2, jaw catching and locking as an affirmative answer to Q3, and TMD as an affirmative answer to any of the 3Q/TMD questions (i.e., OFP and/or jaw catching and locking).

VIP registry data allowed classification of 3 additional common and comparative health states that are likely to negatively affect health-related quality of life: myocardial events, type 1 diabetes, and anxiety. These 3 states have corresponding DWs and lay descriptions provided within the Global Burden of Disease program (Appendix 1). Therefore, it is important to adjust for these variables to isolate the impact of OFP/TMD on overall health-related quality of life. Myocardial events, such as controlled, medically managed heart failure due to ischemic heart disease, were defined by self-reported myocardial infarction in the VIP registry that required hospitalization. Type 1 diabetes was defined according to self-reported information on diabetes in the VIP registry and the use of diabetes medication. Anxiety was defined per self-reported prescription and use of medication for anxiety or sleep disturbances as stated in the VIP registry. Furthermore, self-reported data on history of any sick leave >6 mo were extracted from the VIP registry.

### Characterization of Health States Based on Clinical Data

#### Pain Disability

The pain-related disability of OFP and TMD was assessed by the GCPS, a 7-item questionnaire including measures of pain intensity, interference with daily activities, and number of disability days (Von Korff et al. 1992; Sharma et al. 2021). Characteristic pain intensity (CPI) was evaluated by a numerical rating scale (NRS), ranging from 0 (no pain) to 100 (worst imaginable pain), and presented as the average pain intensity over the past month. In addition, the 7 items were categorized into 5 hierarchical and discrete disability levels (I to IV).

#### Jaw Limitation

The JFLS-20 was used to assess functional limitations of the jaw (Ohrbach et al. 2008). This 20-item questionnaire covers 3 domains: mastication (items 1 to 6), vertical jaw movements (items 7 to 12), and communication (items 13 to 20). Each item was scored on an NRS ranging from 0 (no limitation) to 10 (severe limitation) and presented as a total sum score and score for the individual domain.

#### Pain Catastrophizing

The Pain Catastrophizing Scale, encompassing the 3 domains rumination, magnification, and helplessness, was used to evaluate the level of pain catastrophizing. Each of the 13 items was scored from 0 (not at all) to 4 (all the time). A total sum score was calculated, ranging from 0 to 52.

#### Sleep

A single question, “How do you assess your sleep quality?” was used to evaluate sleep quality, with responses ranging from 0 (very good) to 10 (very bad) on a continuous NRS.

### Statistical Methods

The DWs for OFP and TMD were estimated per the SF-36 (Burstein et al. 2015). One methodological challenge was that the SF-36 (from participation in VIP registry) and OFP/TMD (from dental visits) were assessed at different time points. Each respondent was therefore assigned an OFP/TMD status based on answers to 3Q/TMD from the dental check-up closest in time to the date of completing the SF-36 questionnaire. Following this, 2 cohorts were defined:

Cohort 1: individuals with 3Q/TMD evaluation within 2 y before or after completing the SF-36 questionnaire. If more than one 3Q/TMD evaluation fulfilled this criterion for a participant (e.g., with one 3Q/TMD evaluation the year before the SF-36 questionnaire and one 3Q/TMD evaluation the year after), the latter evaluation was used.
Cohort 2 (as a subset from cohort 1): individuals with 3Q/TMD evaluations in the same year as completion of the SF-36 questionnaire.

As a result, cohort 2 was smaller than cohort 1 but with less potential OFP/TMD status misclassifications because of the shorter period between the SF-36 and 3Q/TMD data collections. Sets of DWs for OFP and TMD were estimated from the SF-36 in cohort 1 and cohort 2 separately, and their results were compared to assess possible bias and statistical uncertainty related to issues of sampling at different time points.

Burstein et al. (2015) mapped self-reported patient health, estimated as the sum of the PCS and the MCS of the SF-12, into a corresponding cumulative DW representing an estimate of one’s total disability. By using their function, cumulative DWs were estimated for each person based on the SF-36 scores across all individuals. In addition, the cumulative DW was stratified by sex to illustrate the sex-specific contribution to the overall disability. The cumulative DWs were used as the dependent variable in a generalized linear model (GLM) with a logit link function, restricting the cumulative DW to a range between 0 and 1. DWs for OFP were estimated by a GLM with binary indicator variables for OFP and jaw catching/locking, separately, as well as for the other comorbid comparator conditions as independent variables. In a separate model, DWs for TMD were estimated with a GLM with a binary indicator variable for TMD, as well as for the comorbid comparator conditions—myocardial event, diabetes, and anxiety—as independent variables for adjustment in the multivariable analyses. Moreover, since OFP and TMD are associated with an increased risk of having a history of sick leave >6 mo, even if the OFP or TMD themselves may not typically be the primary reason for work absence, the analysis was adjusted for history of sick leave as a proxy for residual comorbid conditions for which data were not available. The procedure partitioned the cumulative disability index into condition-specific DWs for each condition, as well as for history of sick leave. The DW for OFP and TMD were separately estimated the mean condition-specific DW across all individuals. Furthermore, to isolate the effect of OFP from that of jaw catching/locking, the latter was examined by a separate model. *DW estimates with 95% CIs were calculated by nonparametric….*

Characteristics and distributions of the clinical data were provided as descriptive statistics. As the sample was population based and its size originally determined for a different research question, there are only a few participants in the more severe groups (GCPS III and IV). Consequently, significance tests comparing characteristics among pain disability groups were deemed inappropriate and not performed.

The analysis was conducted in R and SPSS version 28. Regression models were fitted with the function *glmmTMB* from the R package *glmmTMB*. The study was reviewed and approved by the Regional Ethical Review Board at Umeå University (2012-331-31 and 2018-393-31) and by the Swedish Ethical Review Authority (2024-06491-02). All participants in the clinical examination provided their written consent for participation. The STROBE statement was followed.

## Results

**Figure 1.**
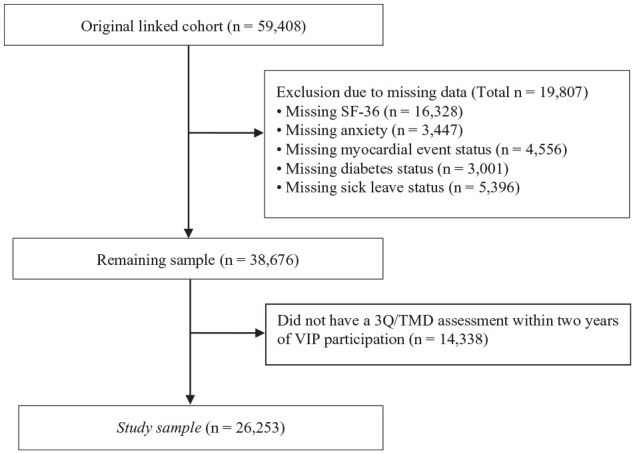
Flowchart of the inclusion process. 3Q/TMD, 3 screening questions in relation to temporomandibular disorder; SF-36, Short Form Health Survey; VIP, Västerbotten Intervention Programme.

**Figure 2.**
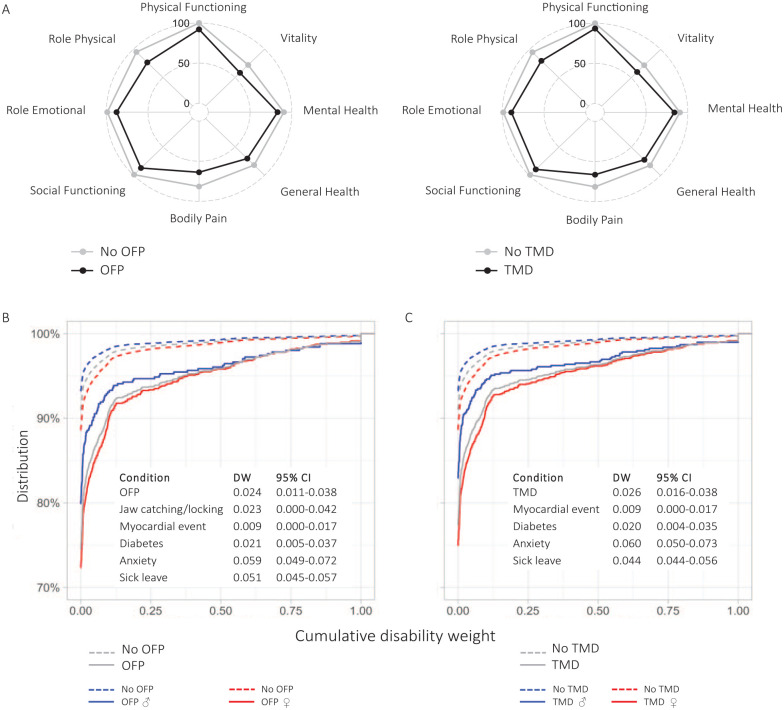
(**A**) Mean values of reported health-related quality of life from the Short Form Health Survey in the different domains for cohort 1 (*n* = 26,253) stratified by orofacial pain (OFP) (*n* = 1,436) and temporomandibular disorders (TMDs; *n* = 1,825). Distribution of the cumulative disability weight (DW) for individuals with and without (**B**) orofacial pain and (**C**) TMD, stratified by sex (women, red lines; men, blue lines). The y-axis is truncated at 70%, since the majority had a cumulative DW of 0. Tables provide information on the adjusted DWs across the total sample.

**Figure 3.**
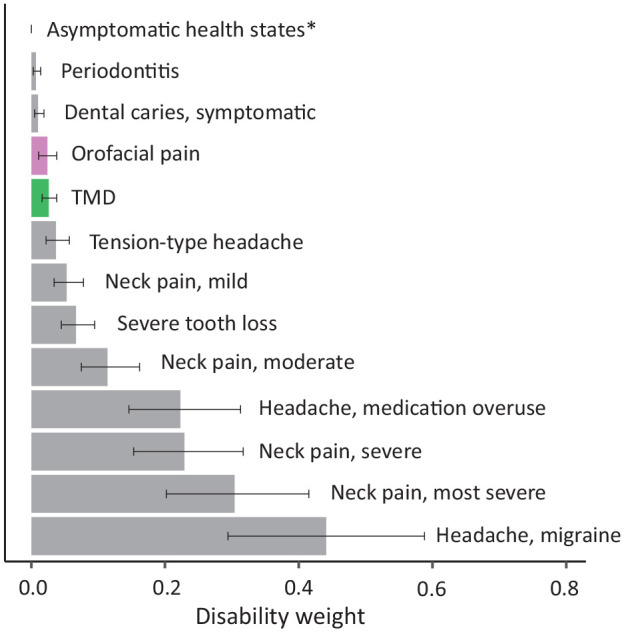
Distribution of disability weights (0 to 1) and 95% CIs for orofacial pain and temporomandibular disorder (TMD) with similar conditions. *All asymptomatic states are set to 0.

The study sample consisted of 26,253 participants (49.9% women; Fig. 1, Table 1). Respondents with OFP reported significantly lower scores on the mental and physical components of the SF-36 (*P* < 0.001), as did those with TMD (*P* < 0.001; Fig. 2A). Furthermore, individuals with OFP and TMD had higher levels of cumulative DW than those without OFP/TMD, reflecting the total disability among persons with OFP/TMD, including other comorbid conditions. Distributions of cumulative DW across the total sample with sex stratifications are presented in Figure 2B and C. Women had higher cumulative DWs as compared with men, albeit with small overall differences. After adjusting for comorbid comparative conditions, the mean DW was 0.024 (95% CI, 0.011 to 0.038) for OFP and 0.026 (95% CI, 0.016 to 0.038) for TMD. The distribution of DWs available for other related health conditions, including periodontitis (DW, 0.007; 95% CI, 0.003 to 0.014) and caries (DW, 0.01; 95% CI, 0.005 to 0.019), is presented in Figure 3 and Appendix Table 2. Corresponding sensitivity analyses based on cohort 2 showed very similar results (Appendix Tables 3 and 4, Appendix Materials).

**Table 1.** Descriptive Characteristics for Cohort 1.

Characteristic	Overall (*N* = 26,253)	Men (*n* = 13,156)	Women (*n* = 13,097)
Age, y	50 (40 to 60)	50 (40 to 60)	50 (40 to 60)
Orofacial pain	1,436 (5.5)	412 (3.1)	1,024 (7.8)
TMD	1,824 (6.9)	558 (4.2)	1,266 (9.7)
Diabetes	1,237 (4.7)	753 (5.7)	484 (3.7)
Myocardial event	665 (2.5)	455 (3.5)	210 (1.6)
Anxiety	1,758 (6.7)	621 (4.7)	1,137 (8.7)
SF-36			
PCS	87 (75 to 94)	88 (77 to 94)	85 (71 to 93)
MCS	87 (76 to 93)	89 (80 to 93)	86 (72 to 92)

Data are presented as median (IQR) or *n* (%).

MCS, Mental Component Summary; PCS, Physical Component Summary; SF-36, Short Form Health Survey; TMD, temporomandibular disorder.

Outcomes from the clinical examinations are presented in Table 2. The mean CPI among individuals with OFP, as categorized by Q1 and/or Q2, was very similar to the mean CPI among those with TMD pain, as diagnosed in the clinical examination (3.9 [SD, 2.2] vs 3.8 [SD, 2.2]). Participants with pain disability and high CPI (GCPS IIa or higher) reported median JFLS-20 scores between 19.0 and 44.0. Moreover, those with higher pain disability (GCPS I or higher) reported higher levels of pain catastrophizing. In addition, respondents with OFP reported a mean sleep quality score that was 1 point lower than those without OFP (5.0 [SD, 2.8] vs 3.81 [SD, 2.6]).

**Table 2.** Orofacial Pain Severity and Related Characteristics in the Subsample with Clinical Data (*N* = 300).

	Graded Chronic Pain Scale			
Characteristic	I (*n* = 116)	IIa (*n* = 32)	IIb (*n* = 7)	III (*n* = 8)
Individuals, *n*				
Orofacial pain	65	28	7	8
TMD	73	29	7	8
Only question 3	8	1	0	0
TMD pain, DC/TMD	65	24	7	8
TMD, DC/TMD	92	28	7	8
Characteristic pain intensity ^a,b^	2.2 (1.3)	5.9 (0.9)	6.7 (0.9)	5.8 (1.5)
Functional jaw limitations ^b,c^	3 (12)	42.0 (58.8)	25 (100)	19.0 (68.8)
Mastication	1 (4)	12 (20.5)	14 (29.0)	10.0 (19.0)
Vertical jaw mobility	1 (4)	9 (16.5)	8 (21.0)	5.0 (15.8)
Emotional and verbal expression	0 (4)	12.5 (22.3)	3 (50.0)	6.0 (34.3)
Pain catastrophizing ^c,d^	7 (14.5)	15.5 (22.8)	16 (28.0)	24.5 (35.5)
Sleep quality ^a^	4.6 (2.5)	5.7 (3.1)	3.6 (3.3)	6.9 (2.6)

Note that Graded Chronic Pain Scale IV is not presented due to data confidentiality and small sample size (*n* = 1) but is included in the analysis.

DC/TMD, Diagnostic Criteria for Temporomandibular Disorders; TMD, temporomandibular disorder.

a Mean (SD).

b From Jaw Functional Limitation Scale–20.

c Median (75% percentile).

d From the Physical Component Summary.

## Discussion

### Key Finding

Our findings indicate that DWs for OFP and TMD are higher than for symptomatic caries and periodontitis alone, highlighting the importance of including OFP in GBD estimates of oral health. In corresponding lay descriptions, key factors such as pain intensity, distress, and functional jaw limitations should be considered with the broader impact on daily life, including effects on sleep quality (John et al. 2020).

At an overall level, our findings support the general understanding of the relationship between OFP, including TMD, and comorbidities and their negative impact on health-related quality of life. In relation to estimates of disease burden and when compared with other diseases, chronic musculoskeletal pain conditions such as TMD are nonmalignant, with the overall burden probably stemming from the high prevalence and chronic nature rather than the mortality. As reflected in the relatively low DWs assigned to oral health conditions, this applies to other oral health issues, such as caries-related pain and edentulism, which affect oral function but have a limited impact on overall disability (Global Burden of Disease Collaborative Network 2020). Our finding of DWs within the range of comparable pain conditions, such as mild neck pain, even after adjusting for some common comorbidities, coupled with the similarity in outcomes from the DEEP study of UK adults (Durham et al. 2015) is reassuring for the validity of our results. Moreover, our estimation of DWs for the factors that we adjust for aligns well with the DWs already implemented in Global Burden of Disease measures for these health states, which further supports the validity of the findings. Given the well-established differences in pain prevalence, including OFP, between men and women, it may be worthwhile to stratify DWs by sex to evaluate whether women and men contribute similarly to the burden of pain.

The importance of recognizing the overall burden of OFP and TMD is evident in the long-standing inclusion of psychosocial assessment as a standard component of classification of OFP (“International Classification of Orofacial Pain” 2020), as well as in the internationally adopted criteria for TMD (Schiffman et al. 2014). As a result, a robust body of evidence, grounded in validated resources, can provide guidance on further characterizing the severity of these conditions. Building on the clinical data available, our findings and previous research on general population samples (Miller et al. 2019) highlight the relationship between pain intensity and pain-related disability. Moreover, pain catastrophizing, a predictor of distress (Severeijns et al. 2001), and functional jaw limitations have consistently been associated with OFP and TMD, even in general population samples (Thomas et al. 2023). Although not clinically significant in our sample, distress, as reflected in the correlation between pain catastrophizing and pain intensity (Häggman-Henrikson et al. 2021), underscores the relationship between pain-related disability and distress. This warrants consideration for inclusion of distress in lay descriptions of OFP severity. Our finding of a significant association between functional jaw limitations and TMD emphasizes the importance of understanding the relationship between jaw function and disability (Miller et al. 2020), especially since studies on the isolated impact of functional oral, jaw, and general limitations on disability remain scarce. Finally, considering the well-established and bidirectional relationship between pain and sleep quality, our findings indicate that sleep quality, as defined by validated questionnaires in appropriate samples, may be a factor to consider in descriptions of OFP severity.

The use of data on health-related quality of life is one of the methods described for estimating DW (Charalampous et al. 2022). Given the substantial evidence of the negative impact on the quality of life for those with OFP (Dahlstrom and Carlsson 2010; Vallin et al. 2024), such a data-driven approach may be considered the most reasonable, as it enables comparisons with already available data. Whether caused by a toothache or chronic TMD, OFP has a similar impact on the person (Shueb et al. 2015), and data utilizing generic health utility measures such as the EQ-5D-5L suggest that the impacts are at least similar to, if not greater than, depression and arthritis (Durham et al. 2015). Providing confirmatory evidence, EQ-5D data on OFP show similar trends as the present study based on the SF-36, thus supporting use of the SF-36, especially for disability estimations for pain conditions.

OFP prevalence varies across populations and regions (Porporatti et al. 2024). Higher prevalence of pain may, in turn, be associated with an increase in overlapping pain conditions and other comorbidities, thereby contributing to disability and consequently overall burden. Our results were derived from a large, Swedish, general population–based cohort, with prevalence numbers of OFP in line with those commonly reported in the literature (Manfredini et al. 2011; Häggman-Henrikson et al. 2020; Vallin et al. 2024). Moreover, prevalence estimates for general pain place Sweden at the median (Zimmer et al. 2022), making general population samples from Sweden representative for averaged estimates. In addition, the SF-36 scores in this cohort were well in line with normative values from general population samples in Sweden (Vallin et al. 2024), supporting the validity of the study findings.

### Limitations

It should be noted that this 3Q/TMD questionnaire cannot differentiate among specific types of conditions (i.e., OFP) but may represent an overall estimate in the orofacial region. Moreover, although the 3Q/TMD was introduced to facilitate the identification of persons with potential TMD, as illustrated by the sensitivity of 0.82 (95% CI, 0.74 to 0.88), other conditions, such as dental pain, may be captured with the questions. Therefore, it was deemed reasonable to classify affirmative answers to Q1 or Q2 as being indicative of OFP. In the present cohort, there was a time lag of up to 2 y between respondents answering the SF-36 survey and the 3Q/TMD questions. However, sensitivity analyses (Appendix Data) based only on participants with SF-36 and 3Q/TMD data from within the same calendar year gave very similar results, indicating the robustness of the findings.

Oral health is by definition regarded as multifaceted and includes many activities central in daily living, such as eating and communicating (FDI World Dental Federation n.d. ; Glick et al. 2017), thus reflecting factors essential to quality of life. In this regard, it is especially important to note that all abilities related to oral health should be possible without pain and discomfort per the *Draft Global Strategy on Oral Health* report of the World Health Organization (2022). It is therefore important to expand global measures on oral health beyond caries and periodontitis and to incorporate OFP, including TMD, in global health measures of disease burden, as recently advocated (Lobbezoo et al. 2022). Although we present DWs for OFP and TMD, a next step would be to categorize each health state, including severity, with a corresponding DW. Future research should focus on linking SF-36 data to high-resolution clinical data to provide more well-defined DWs, taking into account other overlapping pain conditions (e.g., migraine and tension-type headaches), pain severity, and their interactions. In addition, involving panel judges, such as stakeholders, patients, and clinicians, would be valuable for refining and finalizing lay descriptions. Ultimately, applying the proposed DWs alongside standardized assessment methods for prevalence estimates, such as the DC/TMD and the 3Q/TMD, could provide reliable and comparable data on the global burden of OFP and TMD.

## Conclusion

Given the impact of OFP on well-being, our findings demonstrate the importance of including OFP and TMD into the Global Burden of Disease program. Further efforts are needed to develop acceptable lay descriptions and to validate DW findings in other populations.

## Author Contributions

A. Lövgren, contributed to conception, design, data analysis, drafted the manuscript; P. Liv, contributed to conception, design, data analysis, critically revised the manuscript; J. Durham, D.A.G. Goncalves, F.P. Kapos, S. Kothari, M. Drangsholt, C.C. Peck, C.M. Visscher, L. Ong, P. Svensson, contributed to conception and design, critically revised the manuscript. All authors gave final approval and agree to be accountable for all aspects of the work.

## Supplemental Material

sj-docx-1-jdr-10.1177_00220345251363852 – Supplemental material for Disability Weights for Global Burden Estimation of Orofacial Pain Supplemental material, sj-docx-1-jdr-10.1177_00220345251363852 for Disability Weights for Global Burden Estimation of Orofacial Pain by A. Lövgren, P. Liv, J. Durham, D.A.G. Goncalves, F.P. Kapos, S.F. Kothari, M. Drangsholt, C.C. Peck, C.M. Visscher, L. Ong and P. Svensson in Journal of Dental Research

## References

[bibr1-00220345251363852] Breckons M Shen J Bunga J Vale L Durham J. 2018. DEEP study: indirect and out-of-pocket costs of persistent orofacial pain. J Dent Res. 97(11):1200–1206. 30011387 10.1177/0022034518773310

[bibr2-00220345251363852] Burstein R Fleming T Haagsma J Salomon JA Vos T Murray CJ . 2015. Estimating distributions of health state severity for the Global Burden of Disease study. Popul Health Metr. 13:31. doi:10.1186/s12963-015-0064-y 26582970 PMC4650517

[bibr3-00220345251363852] Charalampous P Polinder S Wothge J von der Lippe E Haagsma JA . 2022. A systematic literature review of disability weights measurement studies: evolution of methodological choices. Arch Public Health. 80(1):91. doi:10.1186/s13690-022-00860-z 35331325 PMC8944058

[bibr4-00220345251363852] Dahlström L Carlsson GE . 2010. Temporomandibular disorders and oral health–related quality of life: a systematic review. Acta Odontol Scand. 68(2):80–85. 20141363 10.3109/00016350903431118

[bibr5-00220345251363852] Durham J . 2023. Keynote address: too many decisions: uncertainty in temporomandibular disorders. J Dent Res. 102:0604. https://iadr.abstractarchives.com/abstract/23iags-3882553/keynote-address-too-many-decisions-uncertainty-in-temporomandibular-disorders-tmd.

[bibr6-00220345251363852] Durham J Steele JG Breckons M Story W Vale L. 2015. DEEP study: does EQ-5D-5l measure the impacts of persistent oro-facial pain? J Oral Rehabil. 42(9):643–650. 25818477 10.1111/joor.12296 PMC5609658

[bibr7-00220345251363852] FDI World Dental Federation. n.d. FDI’s definition of oral health. Geneva: FDI World Dental Federation [accessed 2024 Dec 29]. https://www.fdiworlddental.org/fdis-definition-oral-health.

[bibr8-00220345251363852] GBD 2019 Diseases and Injuries Collaborators. 2020. Global burden of 369 diseases and injuries in 204 countries and territories, 1990–2019: a systematic analysis for the Global Burden of Disease Study 2019. Lancet. 396(10258):1204–1222. 33069326 10.1016/S0140-6736(20)30925-9 PMC7567026

[bibr9-00220345251363852] GBD 2021 Low Back Pain Collaborators. 2023. Global, regional, and national burden of low back pain, 1990–2020, its attributable risk factors, and projections to 2050: a systematic analysis of the Global Burden of Disease Study 2021. Lancet Rheumatol. 5(6):e316–e329. doi:10.1016/s2665-9913(23)00098-x PMC10234592 37273833

[bibr10-00220345251363852] Glick M Williams DM Kleinman DV Vujicic M Watt RG Weyant RJ . 2017. A new definition for oral health developed by the FDI World Dental Federation opens the door to a universal definition of oral health. J Public Health Dent. 77(1):3–5. 28276588 10.1111/jphd.12213

[bibr11-00220345251363852] Global Burden of Disease Collaborative Network. 2020. Global Burden of Disease Study 2019 (GBD 2019) disability weights. Seattle (WA): Institute for Health Metrics and Evaluation. doi:10.6069/1W19-VX76

[bibr12-00220345251363852] Häggman-Henrikson B Liv P Ilgunas A Visscher CM Lobbezoo F Durham J Lövgren A. 2020. Increasing gender differences in the prevalence and chronification of orofacial pain in the population. Pain. 161(8):1768–1775. 32701837 10.1097/j.pain.0000000000001872 PMC7365674

[bibr13-00220345251363852] Häggman-Henrikson B Visscher CM Wanman A Ljotsson B Peck C Lövgren A. 2021. Even mild catastrophic thinking is related to pain intensity in individuals with painful temporomandibular disorders. J Oral Rehabil. 48(11):1193–1200. 34462940 10.1111/joor.13251

[bibr14-00220345251363852] International classification of orofacial pain, 1st edition (ICOP). 2020. Cephalalgia. 40(2):129–221. 10.1177/0333102419893823 32103673

[bibr15-00220345251363852] Jenkinson C Layte R Jenkinson D Lawrence K Petersen S Paice C Stradling J. 1997. A shorter form health survey: can the SF-12 replicate results from the SF-36 in longitudinal studies? J Public Health Med. 19(2):179–186. 9243433 10.1093/oxfordjournals.pubmed.a024606

[bibr16-00220345251363852] John MT Sekulic S Bekes K Al-Harthy MH Michelotti A Reissmann DR Nikolovska J Sanivarapu S Lawal FB List T , et al. 2020. Why patients visit dentists—a study in all World Health Organization regions. J Evid Based Dent Pract. 20(3):101459. doi:10.1016/j.jebdp.2020.101459 32921379 PMC7490464

[bibr17-00220345251363852] Kaplan SE Ohrbach R. 2016. Self-report of waking-state oral parafunctional behaviors in the natural environment. J Oral Facial Pain Headache. 30(2):107–119. 27128474 10.11607/ofph.1592

[bibr18-00220345251363852] Lobbezoo F Aarab G Kapos FP Dayo AF Huang Z Koutris M Peres MA Thymi M Haggman-Henrikson B. 2022. The global need for easy and valid assessment tools for orofacial pain. J Dent Res. 101(13):1549–1553. 35883282 10.1177/00220345221110443 PMC9693714

[bibr19-00220345251363852] Lövgren A Karlsson Wirebring L Häggman-Henrikson B Wänman A. 2018. Decision-making in dentistry related to temporomandibular disorders: a 5-yr follow-up study. Eur J Oral Sci. 126(6):493–499. 30298596 10.1111/eos.12572

[bibr20-00220345251363852] Lövgren A Visscher CM Häggman-Henrikson B Lobbezoo F Marklund S Wänman A. 2016. Validity of three screening questions (3Q/TMD) in relation to the DC/TMD. J Oral Rehabil. 43(10):729–736. 27573533 10.1111/joor.12428

[bibr21-00220345251363852] Manfredini D Guarda-Nardini L Winocur E Piccotti F Ahlberg J Lobbezoo F. 2011. Research diagnostic criteria for temporomandibular disorders: a systematic review of axis I epidemiologic findings. Oral Surg Oral Med Oral Pathol Oral Radiol Endod. 112(4):453–462. 21835653 10.1016/j.tripleo.2011.04.021

[bibr22-00220345251363852] Miller VE Chen DG Barrett D Poole C Golightly YM Sanders AE Ohrbach R Greenspan JD Fillingim RB Slade GD . 2020. Understanding the relationship between features associated with pain-related disability in people with painful temporomandibular disorder: an exploratory structural equation modeling approach. Pain. 161(12):2710–2719. 32639367 10.1097/j.pain.0000000000001976 PMC7669591

[bibr23-00220345251363852] Miller VE Poole C Golightly Y Barrett D Chen DG Ohrbach R Greenspan JD Fillingim RB Slade GD . 2019. Characteristics associated with high-impact pain in people with temporomandibular disorder: a cross-sectional study. J Pain. 20(3):288–300. 30292793 10.1016/j.jpain.2018.09.007 PMC6424335

[bibr24-00220345251363852] Norberg M Wall S Boman K Weinehall L. 2010. The Västerbotten Intervention Programme: background, design and implications. Glob Health Action. 3(1):4643. doi:10.3402/gha.v3i0.4643 PMC2844807 20339479

[bibr25-00220345251363852] Ohrbach R Larsson P List T. 2008. The Jaw Functional Limitation Scale: development, reliability, and validity of 8-item and 20-item versions. J Orofac Pain. 22(3):219–230. 18780535

[bibr26-00220345251363852] Pedler A . 2010. The Pain Catastrophising Scale. J Physiother. 56(2):137. doi:10.1016/s1836-9553(10)70047-3 20482485

[bibr27-00220345251363852] Porporatti AL Schroder AGD Lebel A Moreau N Guillouet C Stechman-Neto J Boucher Y. 2024. Prevalence of orofacial and head pain: an umbrella review of systematic reviews. J Oral Facial Pain Headache. 38(3):1–14. 10.22514/jofph.2024.022 PMC11810662 39800567

[bibr28-00220345251363852] Salinas Fredricson A Kruger Weiner C Adami J Rosen A Lund B Hedenberg-Magnusson B Fredriksson L Svedberg P Naimi-Akbar A . 2023. Sick leave and disability pension among TMD patients with musculoskeletal diseases, mental and behavioural disorders—a SWEREG-TMD population-based cohort study. BMC Public Health. 23(1):852. doi:10.1186/s12889-023-15815-4 37165335 PMC10173494

[bibr29-00220345251363852] Schiffman E Ohrbach R Truelove E Look J Anderson G Goulet JP List T Svensson P Gonzalez Y Lobbezoo F , et al. 2014. Diagnostic Criteria for Temporomandibular Disorders (DC/TMD) for clinical and research applications: recommendations of the International RDC/TMD Consortium Network and Orofacial Pain Special Interest Group. J Oral Facial Pain Headache. 28(1):6–27. 24482784 10.11607/jop.1151 PMC4478082

[bibr30-00220345251363852] Severeijns R Vlaeyen JW van den Hout MA Weber WE . 2001. Pain catastrophizing predicts pain intensity, disability, and psychological distress independent of the level of physical impairment. Clin J Pain. 17(2):165–172. 11444718 10.1097/00002508-200106000-00009

[bibr31-00220345251363852] Sharma S Kallen MA Ohrbach R. 2021. Graded Chronic Pain Scale: validation of 1-month reference frame. Clin J Pain. 38(2):119–131. 34803153 10.1097/AJP.0000000000001005 PMC8727576

[bibr32-00220345251363852] Shueb SS Nixdorf DR John MT Alonso BF Durham J. 2015. What is the impact of acute and chronic orofacial pain on quality of life? J Dent. 43(10):1203–1210. 26073033 10.1016/j.jdent.2015.06.001

[bibr33-00220345251363852] Slade G Durham J. 2020. Appendix C: prevalence, impact, and costs of treatment for temporomandibular disorders. In: Yost O Liverman CT English R Mackey S Bond EC , editors. Temporomandibular disorders: priorities for research and care. Washington (DC): National Academies Press. 32200600

[bibr34-00220345251363852] Thomas S Wang Y Cundiff-O’Sullivan R Massalee R Colloca L. 2023. How negative and positive constructs and comorbid conditions contribute to disability in chronic orofacial pain. Eur J Pain. 27(1):99–110. 36203350 10.1002/ejp.2042 PMC9799734

[bibr35-00220345251363852] Vallin S Liv P Haggman-Henrikson B Visscher CM Lobbezoo F Lovgren A. 2024. Temporomandibular disorder pain is associated with increased sick leave and reduced health related quality of life. Eur J Pain. 28(10):1827–1840. 39072933 10.1002/ejp.2314

[bibr36-00220345251363852] Von Korff M Ormel J Keefe FJ Dworkin SF . 1992. Grading the severity of chronic pain. Pain. 50(2):133–149. 1408309 10.1016/0304-3959(92)90154-4

[bibr37-00220345251363852] Ware JE Jr Sherbourne CD . 1992. The MOS 36-item Short-Form Health Survey (SF-36). I: Conceptual framework and item selection. Med Care. 30(6):473–483. 1593914

[bibr38-00220345251363852] World Health Organization. 2022. Draft global strategy on oral health. Geneva: World Health Organization [accessed 2025 Jan 31]. https://apps.who.int/gb/ebwha/pdf_files/WHA75/A75_10Add1-en.pdf.

[bibr39-00220345251363852] Zimmer Z Fraser K Grol-Prokopczyk H Zajacova A. 2022. A global study of pain prevalence across 52 countries: examining the role of country-level contextual factors. Pain. 163(9):1740–1750. 35027516 10.1097/j.pain.0000000000002557 PMC9198107

