# Supplementary material for: Disability Weights for Global Burden Estimation of Orofacial Pain
Source: J Dent Res. 2025 Sep 15;105(4):468–75. doi: 10.1177/00220345251363852 (PMC12957409; doi:10.1177/00220345251363852)
Supplement: sj-docx-1-jdr-10.1177_00220345251363852 – Supplemental material for Disability Weights for Global Burden Estimation of Orofacial Pain [file sj-docx-1-jdr-10.1177_00220345251363852.docx]

# Table S1. Disability weights included in the Global Burden of Disease program for common and comparative health states adjusted for in this study [1].

| Sequela | Health state name | Health state lay description | Disability Weight |
| --- | --- | --- | --- |
| Mild heart failure due to ischemic heart disease | Heart failure, mild | is short of breath and easily tires with moderate physical activity, such as walking uphill or more than a quarter-mile on level ground. The person feels comfortable at rest or during activities requiring less effort. | 0.041 (0.026–0.062) |
| Moderate heart failure due to ischemic heart disease | Heart failure, moderate | is short of breath and easily tires with minimal physical activity, such as walking only a short distance. The person feels comfortable at rest but avoids moderate activity. | 0.072 (0.047–0.103) |
| Uncomplicated diabetes mellitus type 1 | Generic uncomplicated disease: worry and daily medication | has a chronic disease that requires medication every day and causes some worry but minimal interference with daily activities. | 0.049 (0.031–0.072) |
| Mild other mental disorders | Anxiety disorders, mild | feels mildly anxious and worried, which makes it slightly difficult to concentrate, remember things, and sleep. The person tires easily but is able to perform daily activities. | 0.03 (0.018–0.046) |
| Moderate other mental disorders | Anxiety disorders, moderate | feels anxious and worried, which makes it difficult to concentrate, remember things, and sleep. The person tires easily and finds it difficult to perform daily activities. | 0.133 (0.091–0.186) |

Table S2. List of states related to TMD and orofacial pain and the corresponding disability weight according to estimates from the 2019 Global Burden of Disease Study [1].

| Sequela | Health state name | Health state lay description | Disability weight |
| --- | --- | --- | --- |
| Pain due to caries of deciduous teeth | Dental caries, symptomatic | has a toothache, which causes some difficulty in eating. | 0.01 (0.005–0.019) |
| Asymptomatic caries of deciduous teeth | Asymptomatic |  | 0 (0–0) |
| Chronic periodontal diseases | Periodontitis | has minor bleeding of the gums from time to time, with mild discomfort. | 0.007 (0.003–0.014) |
| Difficulty eating due to edentulism and severe tooth loss | Severe tooth loss | has lost more than 20 teeth including front and back, and has great difficulty in eating meat, fruits, and vegetables. | 0.067 (0.045–0.095) |
| Mild neck pain | Neck pain, mild | has neck pain, and has difficulty turning the head and lifting things. | 0.053 (0.034–0.078) |
| Moderate neck pain | Neck pain, moderate | has constant neck pain, and has difficulty turning the head, holding arms up, and lifting things | 0.114 (0.075–0.162) |
| Severe neck pain | Neck pain, severe | has severe neck pain, and difficulty turning the head and lifting things. The person gets headaches and arm pain, sleeps poorly, and feels tired and worried. | 0.229 (0.153–0.317) |
| Most severe neck pain | Neck pain, most severe | has constant neck pain and arm pain, and difficulty turning the head, holding arms up, and lifting things. The person gets headaches, sleeps poorly, and feels tired and worried. | 0.304 (0.202–0.415) |
| Symptomatic medication overuse headache due to migraine | Headache, medication overuse | has daily headaches, felt as dull pain and often lasting all day, with poor sleep, nausea and fatigue. The person takes medicine for the headaches, which provides little relief but is needed to avoid having worse symptoms. | 0.223 (0.146–0.313) |
| Asymptomatic medication overuse headache due to migraine | Asymptomatic |  | 0 (0–0) |
| Symptomatic probable migraine | Headache, migraine | has severe, throbbing head pain and nausea that cause great difficulty in daily activities and sometimes confine the person to bed. Moving around, light, and noise make it worse. | 0.441 (0.294–0.588) |
| Symptomatic definite migraine | Headache, migraine | has severe, throbbing head pain and nausea that cause great difficulty in daily activities and sometimes confine the person to bed. Moving around, light, and noise make it worse. | 0.441 (0.294–0.588) |
| Asymptomatic probable migraine | Asymptomatic |  | 0 (0–0) |
| Asymptomatic definite migraine | Asymptomatic |  | 0 (0–0) |
| Symptomatic medication overuse headache due to tension-type headache | Headache, medication overuse | has daily headaches, felt as dull pain and often lasting all day, with poor sleep, nausea and fatigue. The person takes medicine for the headaches, which provides little relief but is needed to avoid having worse symptoms. | 0.223 (0.146–0.313) |
| Asymptomatic medication overuse headache due to tension-type headache | Asymptomatic |  | 0 (0–0) |
| Symptomatic probable tension-type headache | Headache, tension-type | has a moderate headache that also affects the neck, which causes difficulty in daily activities. | 0.037 (0.022–0.057) |
| Symptomatic definite tension-type headache | Headache, tension-type | has a moderate headache that also affects the neck, which causes difficulty in daily activities. | 0.037 (0.022–0.057) |
| Asymptomatic probable tension-type headache | Asymptomatic |  | 0 (0–0) |
| Asymptomatic definite tension-type headache | Asymptomatic |  | 0 (0–0) |

# Table S3. Disability weights for orofacial pain and comorbidities based on Cohort 2

| Disease | DW | 95% CI |
| --- | --- | --- |
| OFP | 0.030 | 0.008–0.046 |
| Jaw catching/locking | 0.025 | 0.000–0.048 |
| Myocardial event | 0.007 | 0.000–0.024 |
| Diabetes | 0.014 | 0.000–0.044 |
| Anxiety | 0.063 | 0.046–0.082 |
| Sick leave | 0.050 | 0.041–0.057 |

# Table S4. Disability weights for temporomandibular disorder and comorbidities based on Cohort 2

| Disease | DW | 95% CI |
| --- | --- | --- |
| TMD | 0.027 | 0.008–0.041 |
| Myocardial event | 0.006 | 0.000–0.023 |
| Diabetes | 0.013 | 0.000–0.036 |
| Anxiety | 0.064 | 0.047–0.081 |
| Sick leave | 0.050 | 0.041–0.058 |

[1] Global Burden of Disease Study 2019 (GBD 2019) Disability Weights.: Seattle, United States of America: Institute for Health Metrics and Evaluation (IHME). 2020.
